# Malignant gastrointestinal melanoma with an unknown primary

**DOI:** 10.4103/0971-5851.60055

**Published:** 2009

**Authors:** M.V.T. Krishna Mohan, Senthil J. Rajappa, T. Vamshidhar Reddy, T. Roshni Paul

**Affiliations:** *Department of Medical Oncology, Nizam's Institute of Medical Sciences, Punjagutta, Hyderabad, AP, India*; 1 *Department of Gastro-enterology, Nizam's Institute of Medical Sciences, Punjagutta, Hyderabad, AP, India*; 2 *Department of Pathology, Nizam's Institute of Medical Sciences, Punjagutta, Hyderabad, AP, India*

**Keywords:** *Gastrointestinal*, *melanoma*, *unknown primary*

## Abstract

Malignant melanoma is rare in India; melanoma presenting as a metastatic disease with an unknown primary, involving the gastrointestinal tract without involving lymph nodes is extremely uncommon. We report a case of a 28-year-old male with a malignant melanoma metastasizing to stomach and liver with an unknown primary. Relevant literature is being reviewed.

## INTRODUCTION

Malignant melanoma is a rare tumor in India with a reported incidence of 0.2 per 100,000 in females and 0.5 per 100,000 in males.[1] Malignant melanoma presenting as a metastatic disease with an unknown primary is uncommon. [2]Melanoma with an unknown primary metastasizing to the viscera without involving lymph nodes is extremely rare in all the reported series. There are various theories explaining the presentation of melanoma with an “unknown primary.” The existence of primary gastrointestinal melanomas is controversial and has been extensively discussed and debated. We report a rare case of a malignant melanoma from an unknown primary site, metastasizing to the stomach and liver.

## CASE REPORT

A 28-year-old male presented with complaints of abdominal pain, anorexia, and weight loss of a month's duration. He had no other complaints. His past history and family history were unremarkable. He had no other comorbidities. The general physical examination was unremarkable. There were no pigmented lesions or scars on the skin, anal canal, and buccal mucosa. An abdominal examination revealed massive nodular, nontender hepatomegaly occupying almost the whole of the abdomen. No splenomegaly or ascites were present. An examination of the cardiovascular, respiratory, and nervous system, and direct ophthalmoscopy were normal.

The complete blood counts, and liver and renal function tests were normal. A contrast-enhanced computerized tomography (CT) scan of the abdomen showed marked hepatomegaly with multiple hypodense lesions in both lobes of the liver. Gastroduodenoscopy revealed two round elevated lesions with surface ulceration and black base-one over the lesser curvature and another over the greater curvature. [1]Two other tiny black-colored flat spots were seen over the mucosa of the body.

A biopsy obtained from the gastric lesion showed solid nests of large polygonal cells in the submucosa with hyperchromatic nuclei and occasional nucleoli with a deposition of a brownish black pigment within the cells suggestive of a malignant melanoma. The surrounding gastric mucosa was normal. [2]Immunohistochemistry with HMB-45 showed cytoplasmic positivity in the tumor cells. [3]

The patient was prescribed Temozolamide 250 mg, orally, once daily for 5 days; he took the first cycle but subsequently had progressive liver failure and succumbed.

## DISCUSSION

The incidence of a metastatic malignant melanoma with an unknown primary (MUP) varies from 2% to 9% in various series. In a large series reported by Armando *et al.,* the incidence of an unknown primary melanoma was 5.6% (55 out of 980 cases). For all the patients with an MUP, the most common site of metastasis was lymph nodes (61%) and the remaining had a disseminated disease. [2]Bowel and liver metastasis was reported only in four cases (7%). In another series of 40 patients with an MUP, 65% had metastases to lymph nodes alone; 28% had visceral lesions. [3]An incidence of 2.6% for MUP has been reported in another series with lymph nodes being the most common site of metastasis. [4]In all the reported series, the incidence of visceral mets alone in MUP is quite uncommon. Several possible etiologies explaining MUP have been postulated: (1) antecedent, unrecognized, spontaneously regressed primary melanoma is the most widely accepted theory; (2) previously excised and histologically misdiagnosed lesion; (3) concurrent unrecognized melanoma; and (4) *de novo* malignant transformation of sequestrated melanocytes. Our patient did not give history of any surgeries in the past and a thorough clinical examination did not reveal any lesions suggestive of a melanoma.

The possibility of a primary gastrointestinal (GI) melanoma was also considered. But the histopathological examination of the gastric biopsy was not consistent with the same. Primary gastrointestinal melanomas are recognized histologically by *in situ* changes in the overlying and adjacent GI epithelium depicting atypical melanocytic cells in the basal layer of the epithelium and extension in a “pagetoid” fashion into the more superficial epithelium; such features were not present in the present case. Moreover, esophagus and anorectum are considered as the most frequent sites for the primary GI melanomas and their existence in the rest of the GI tract is debated. [5–7]However, considering the fact that the diagnosis was made based on a biopsy which could have missed the features of a primary GI melanoma described above, we cannot completely rule out the possibility of a primary in the stomach.

The prognosis and overall survival of patients with an MUP in all reported series has been similar to patients with a known primary in the same stage. [2]However, some series reported a better OS for patients with an MUP with lymph node metastases alone as compared to patients with a known primary and lymph node disease. [3]Patients with GI and/or liver metastases usually have disseminated disease and the reported median survival is between 2 and 4 months. [8]

This case illustrates the fact that stomach can be one of the uncommon sites of metastasis for a malignant melanoma. A melanoma should be considered as one of the rare differential diagnoses in a patient who presents with metastasis of an unknown origin to the liver.

**Figure 1:**
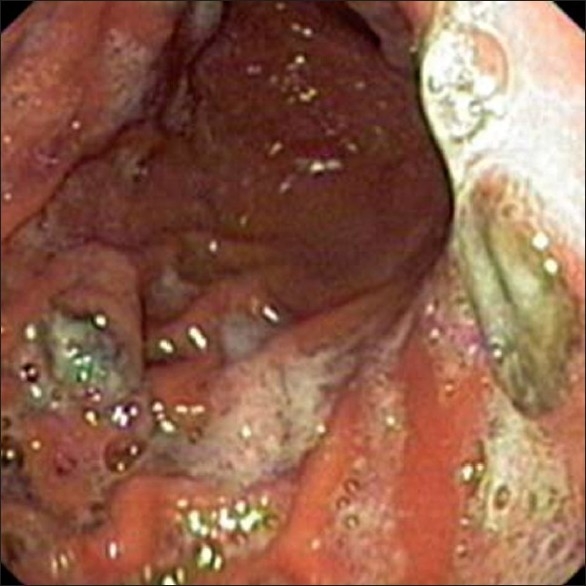
Gastroscopy showing elevated lesions in the stomach with a black base and surface ulceration

**Figure 2:**
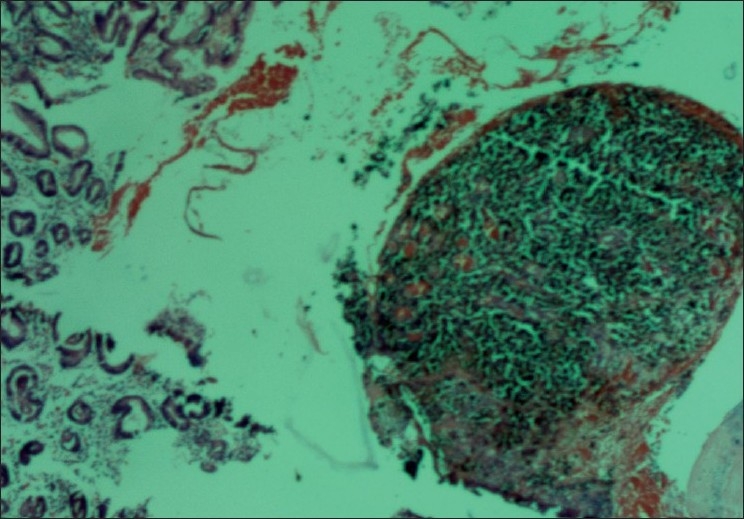
Gastric biopsy showing a malignant melanoma with a normal surrounding mucosa

**Figure 3:**
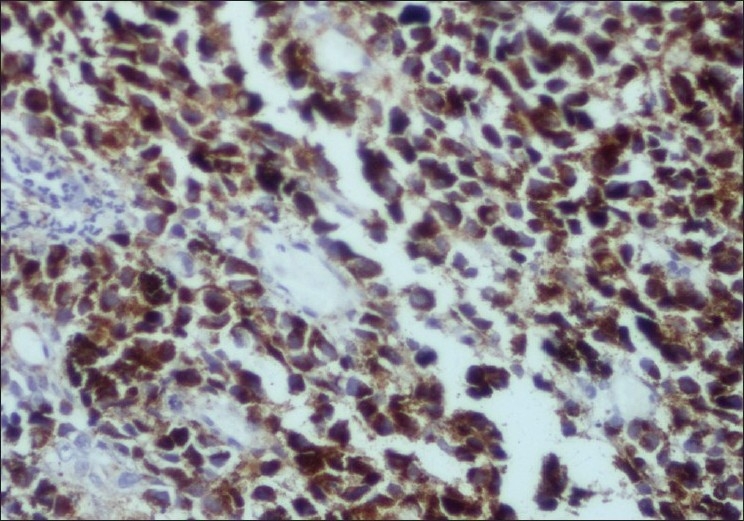
Immunohistochemistry on the biopsy tissue showing cytoplasmic positivity with HMB-45.
